# Discovery of protein biomarkers for venous thromboembolism in non-small cell lung cancer patients through data-independent acquisition mass spectrometry

**DOI:** 10.3389/fonc.2023.1079719

**Published:** 2023-02-09

**Authors:** Yanhong Liu, Lan Gao, Yanru Fan, Rufei Ma, Yunxia An, Guanghui Chen, Yan Xie

**Affiliations:** ^1^ Department of Laboratory Medcine, Henan Provincial People’s Hospital, People’s Hospital of Zhengzhou University, and People’s Hospital of Henan University, Zhengzhou, Henan, China; ^2^ Department of Respiratory, Henan Provincial People’s Hospital, People’s Hospital of Zhengzhou University, and People’s Hospital of Henan University, Zhengzhou, China

**Keywords:** thromboembolism, NSCLC, biomarkers, proteomics, mass spectrometry

## Abstract

**Objective:**

Non-small cell lung cancer (NSCLC) patients present a high incidence of venous thromboembolism (VTE) with poor prognosis. It is crucial to identify and diagnose VTE early. The study aimed to identify potential protein biomarkers and mechanism of VTE in NSCLC patients *via* proteomics research.

**Methods:**

Proteomic analysis of the human plasma was performed through data-independent acquisition mass spectrometry for 20 NSCLC patients with VTE, and 15 NSCLC patients without VTE. Significantly differentially expressed proteins were analyzed by multiple bioinformatics method for further biomarker analysis.

**Results:**

A total of 280 differentially expressed proteins were identified in VTE and non-VTE patients, where 42 were upregulated and 238 were downregulated. These proteins were involved in acute-phase response, cytokine production, neutrophil migration and other biological processes related to VTE and inflammation. Five proteins including SAA1, S100A8, LBP, HP and LDHB had significant change between VTE and non-VTE patients, with the area under the curve (AUC) were 0.8067, 0.8308, 0.7767, 0.8021, 0.8533, respectively.

**Conclusions:**

SAA1, S100A8, LBP, HP and LDHB may serve as potential plasma biomarkers for diagnosis VTE in NSCLC patients.

## 1 Introduction

Venous thromboembolism (VTE), comprising deep vein thrombosis (DVT) and pulmonary embolism (PE), is the common complications and a leading cause of death in patients with cancer (1). The risk of VTE is 4-7 fold higher in patients with cancer than in those without cancer (2, 3). Different cancer types carry different VTE risk (4). Lung cancer is the most common malignancy worldwide and is well established to be high risk of VTE, which account up to 21% of cancer-associated thrombotic cases (5, 6). Non-small cell lung cancer (NSCLC) accounts for more than 80% of lung cancer, with the reported incidence of DVT 13.6% and that of PE to 3.7% (7, 8). The incidence of VTE is higher in patients with NSCLC compared to patients with small cell lung cancer (SCLC) (9).

The diagnosis of VTE mainly depends on ultrasound, venography or pulmonary angiography owing to the lack of typical clinical signs and symptoms, but their costs, invasiveness and subjectivity limit the use in clinical practice. VTE can be ruled out based on a negative D-dimer test result. However, D-dimer may increase in case of cancer, inflammation, infection or necrosis which lead to a poor positive predictive. Cancer patients benefit less from D-dimer because of the high false positive rate (10, 11). Therefore, it is necessary to identify novel biomarkers of VTE in cancer patient.

Plasma is the most easily accessible specimen for the discovery of novel biomarkers. Proteins are usually the final executors of function and involved in extensive biological processes. The integrated effects of congenital and acquired factors that influence the risk of thrombosis are reflected in the protein profile in plasma. A variety of plasma proteins, especially those related to inflammation and fibrinolysis, have been confirmed to be associated with VTE (12, 13). Few studies have investigated the proteomics characteristics or associated signaling pathway of VTE in cancer patient.

Data-independent acquisition mass spectrometry (DIA-MS) is an acquisition scheme of tandem mass spectrometry that is independent of the composition of precursor ions for their fragmentation. It can be combined with the data analysis of proteomics research and has been widely used in the mechanism of disease research (14).

DIA-MS showed high reliability and reproducibility in the screening of novel biomarker in previous studies including in VTE patients (15–17). However, few studies have explored the proteomics features of cancer associated thrombosis. Our study is the first to employ proteomics strategy to identify novel biomarkers for VTE in NSCLC patients. In this study, DIA-MS was used to screen differentially expressed proteins between NSCLC patients with and without VTE to find candidate biomarker for subsequent verification. Then cluster analysis of differentially expressed proteins was used to reveal the specific changes of the biological process.

## 2 Materials and methods

### 2.1 Clinical samples

The study was a case-control study. 20 patients diagnosed with NSCLC within 3 years and VTE within 3 month recruited from Henan Provincial Hospital from 2020 to 2021 were case group (VTE group). 15 patients diagnosed with NSCLC within 3 years but without VTE were classified as control group (non-VTE group). The diagnosis of VTE and NSCLC were confirmed according to the recent guidelines (18–20). The demographic and relevant clinical information were collected.

The blood collection time of VTE group was within 3 month after the diagnosis of VTE, and were collected after treatment with subcutaneous low molecular weight heparin (LMWH). Samples from the non-VTE group were collected after ultrasonic showed no thrombosis. Peripheral blood samples were collected from each participant into tubes containing 3.2% sodium citrate as the anticoagulant and were centrifuged at 1,500 g for 10 minutes to separate plasma. All plasma samples have no jaundice or hemolysis. Samples were stored at -80°C until testing. This study was approved by the Research Ethics Committee of Henan Provincial People’s Hospital.

### 2.2 Mass spectrometry assay for DIA

**Figure 1:**
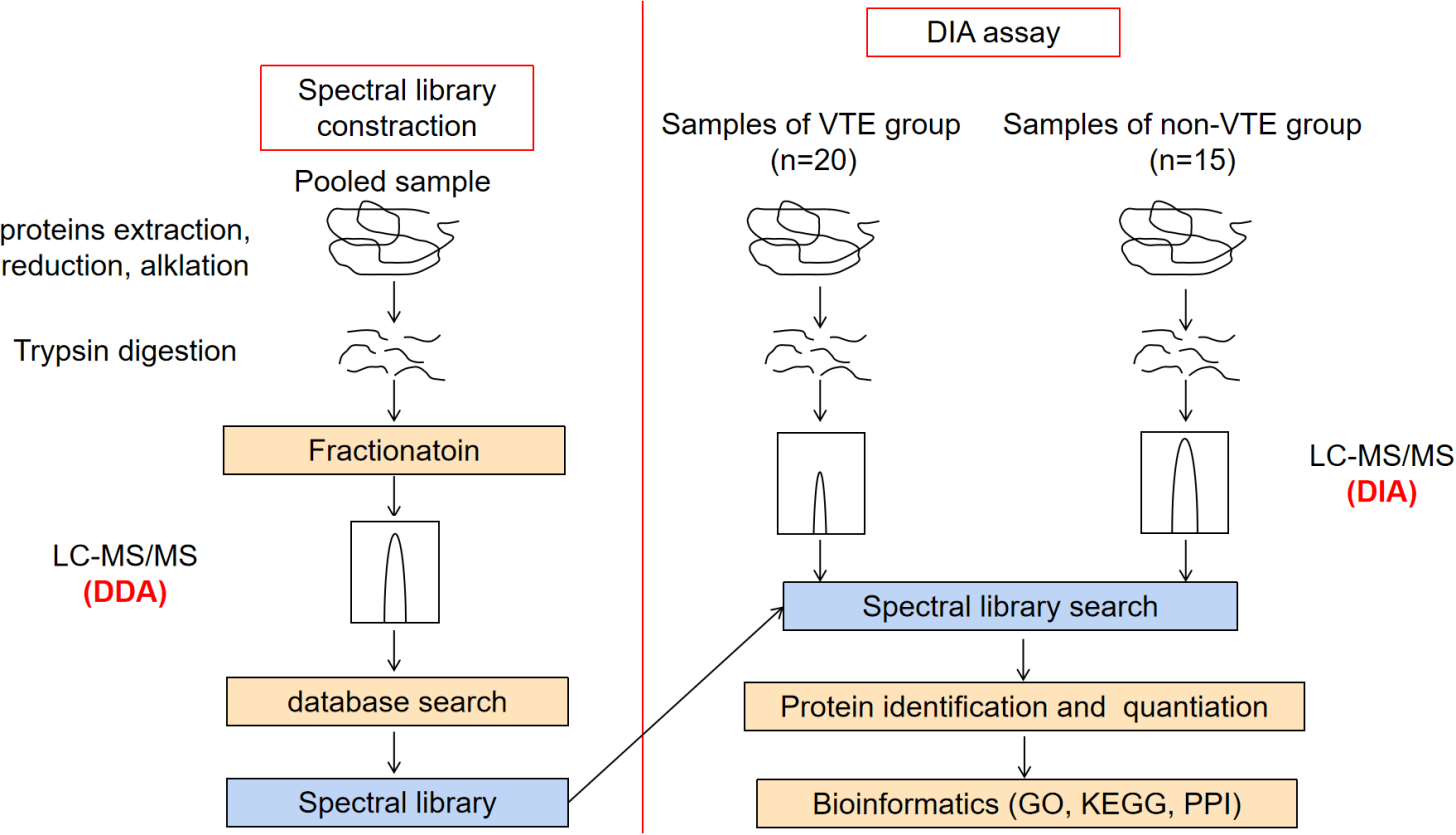
Flowchart of the experiment.

Three main steps were carried out as follows: protein extraction and peptide digestion, data dependent acquisition (DDA) mass spectrometry assay, DIA mass spectrometry assay and data analysis. The workflow is shown in **Figure 1**.

Plasma pools were separated of most abundant proteins following the manufacturer’s protocol (Agilent Technologies). The high and low abundance proteins were collected, desalted and concentrated respectively. 200 μg proteins was repeat ultrafiltered using UA buffer (8 M urea, 150 mM Tris-HCl pH 8.0). Then 100 μl iodoacetamide (100 mM IAA in UA buffer) was added to block reduced cysteine residues and the samples were incubated for 30 min in darkness. The protein suspensions were digested with 4 μg trypsin in 40 μl 25mM NH_4_HCO_3_ buffer overnight at 37°C. Collected peptides were desalted on C18 Cartridges and reconstituted in 40µl of 0.1% formic acid. The iRT-Kits (Biognosys) was added to correct the relative retention time.

All fractions for DDA library generation were analyzed by a Thermo Scientific Q Exactive HF X mass spectrometer connected to an Easy nLC 1200 chromatography system (Thermo Scientific). MS detection method was positive ion, the scan range was 300-1800 m/z, resolution for MS1 scan was 60000 at 200 m/z. Resolution for MS2 scan was 15000. Each DIA cycle contained one full MS-SIM scan, and 30 DIA scans covered a mass range of 350-1800 m/z. QC samples were injected with DIA mode at the beginning of the MS study and after every 6 injections throughout the experiment, which was used to monitor the MS performance.

For DDA library data, the FASTA sequence database was searched with Spectronaut™ software. Spectral library was constructed by importing the original raw files and DDA searching results into Spectronaut Pulsar X™ (Biognosys). DIA data was analyzed with Spectronaut™ 14.4.200727.47784 searching the above constructed spectral library. All results were filtered based on Q value cutoff 0.01 (equivalent to FDR<1%).

### 2.3 Bioinformatic analysis

The differentially expressed proteins were analyzed by gene ontology (GO) and Kyoto Encyclopedia of Genes and Genomes (KEGG) analysis.

The GO terms were mapped and sequences were annotated using the software program Blast2GO. The GO annotation results were plotted by R scripts. The studied proteins were blasted against the online KEGG database to retrieve their KEGG orthology identifications and were subsequently mapped to pathways in KEGG. Enrichment analysis were applied based on the Fisher’ exact test. Benjamini-Hochberg correction for multiple testing was further applied to adjust derived *P* values. And only functional categories and pathways with *P* values under a threshold of 0.05 were considered as significant.

The protein–protein interaction (PPI) information of the studied proteins was retrieved from IntAct molecular interaction database (http://www.ebi.ac.uk/intact/) by their gene symbols or STRING software (http://string-db.org/).

### 2.4 Statistical analysis

For clinical data, Shapiro-Wilk test was performed for normality. Normally distributed data were expressed as the means ± standard deviations. Two independent sample t test was used for statistical analysis and *P* value < 0.05 were considered statistically significant. Non-normally distributed data were reported as medians (interquartile range), and compared using the Mann-Whitney test. Categorical variables were described by percentages and compared using Fisher’s test. Analyses were performed using the SPSS 20.0 software.

For proteomics data, the differentially expressed proteins were identified using the two samples t-distributed test (*P*-value <0.05). The proteins were defined as differentially expressed if the fold change between VTE and non-VTE group was >1.5 or < 0.67 and *P* value < 0.05. The diagnostic value of differentially expressed proteins was evaluated by receiver operating characteristic (ROC) curves and the area under the curve (AUC) was calculated of differentially expressed proteins using GraphPad Prism 7.0.

## 3 Results

### 3.1 Clinical data analysis

20 VTE patients and 15 non-VTE patients with NSCLC were recruited. In VTE group, 13 patients had DVT, 4 patients had PE, 3 patients had both VTE and PE. The age, sex, BMI, comorbidities and clinical test results of cases and controls are summarized in **Table 1**. There were no significant differences in age, sex and BMI between the two groups. PDW and NLR in VTE group were significantly higher than those in non-VTE group (*P*<0.05). However, RBC and HGB in VTE group were significantly lower than those in non-VTE group (*P*<0.05).

**Table 1:** Baseline clinical characteristics.

	VTE group n=20	Non-VTE group n=15	*P*
Age, median (Q1, Q3)	65.50 (55.25,73.00)	66 (58.00,71.00)	0.993^a^
Male/Female (n/n)	9/11	7/8	0.999^b^
BMI, mean ± SD	22.69 ± 3.73	23.88 ± 2.93	0.358^c^
Adenocarcinoma/Non-adenocarcinoma (n/n)	5/15	3/12	0.999^b^
Metastasis/Non-metastasis (n/n)	6/14	4/11	0.999^b^
Comorbidities n (%)			
Hypertension	2 (10.0)	4 (26.7)	0.367^b^
Myocardial infarction	1 (5.0)	0	0.999^b^
Cerebral infarction	2 (10.0)	1 (6.7)	0.999^b^
Diabetes	2 (10.0)	3 (20.0)	0.631^b^
Pneumonia	6 (30.0)	3 (20.0)	0.700^b^
COPD	2 (10.0)	1 (6.7)	0.999^b^
Laboratory Measurements			
WBC, ×10^9^/L, median (Q1, Q3)	5.80 (4.52, 7.92)	5.14 (4.22, 7.12)	0.382^a^
NLR, median (Q1, Q3)	5.95 (3.32, 12.84)	2.40 (1.77, 3.66)	0.002^a^
RBC (×10^12^/L), mean ± SD	3.49 ± 0.53	3.91 ± 0.50	0.023^c^
RDW (fL), mean ± SD	52.05 ± 7.88	47.13 ± 7.10	0.066^c^
HGB (g/L), median (Q1, Q3)	98.00 (88.50, 118.25)	123.00 (114.00, 132.0)	0.018^a^
PLT (×10^9^/L), mean ± SD	197.75 ± 113.65	230.07 ± 52.02	0.315^c^
PDW (fL), median (Q1, Q3)	12.20 (10.58, 15.13)	10.20 (9.50, 11.40)	0.002^a^

BMI, body mass index; WBC, white blood cell; NLR, neutrophil to lymphocyte ratio; RBC, red blood cell; RDW, red blood cell distribution width; HGB, hemoglobin; PLT, platelet; PDW, platelet distribution width.

a Mann-Whitney test

b Fisher’s test

c t test

### 3.2 Proteomics analysis

**Figure 2:**
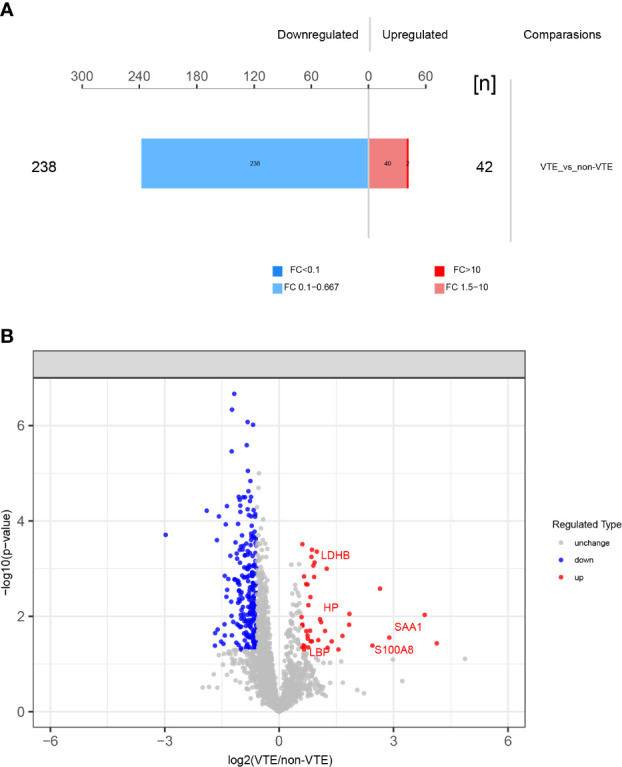
Differentially expressed proteins identified. **(A)** 280 differentially expressed proteins between VTE group and non-VTE group were screened. **(B)** Volcano plot of proteins identified. Red ones stand for the upregulated and blue ones for downregulated proteins.

A total of 3275 proteins and 9007 peptides were identified. We screened 280 differentially expressed proteins. There were 42 upregulated and 238 downregulated proteins in VTE group compared to those in non-VTE group. The total number of differentially expressed proteins and volcano plot of proteins identified in DIA-MS are shown in **Figure 2**.

### 3.3 Differentially expressed proteins analysis

**Figure 3:**
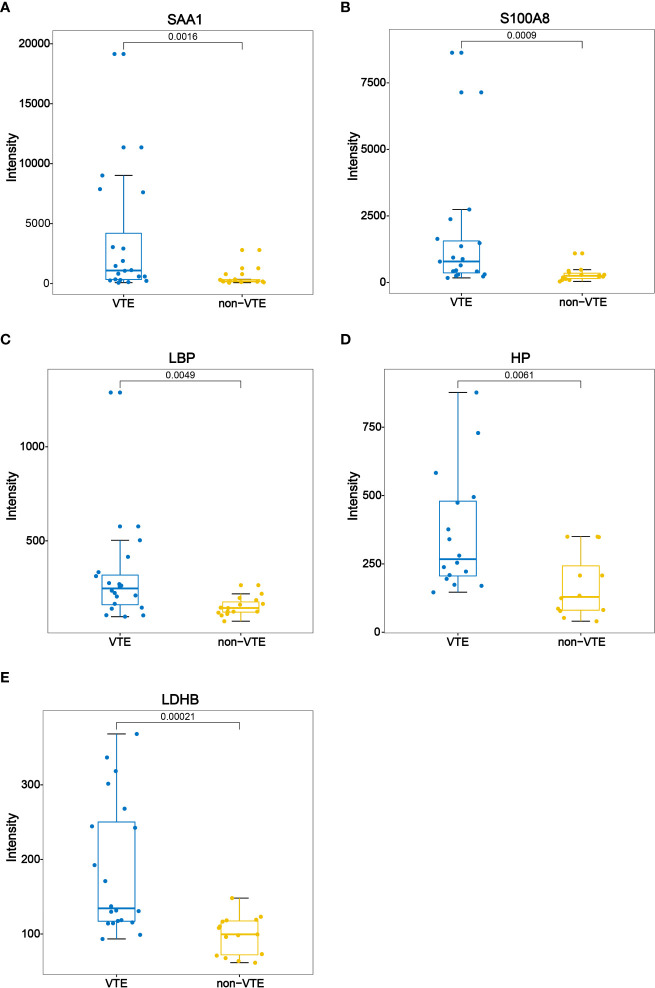
Boxplot of SAA1, S100A8, LBP, HP, LDHB between VTE group and non-VTE group.

**Figure 4:**
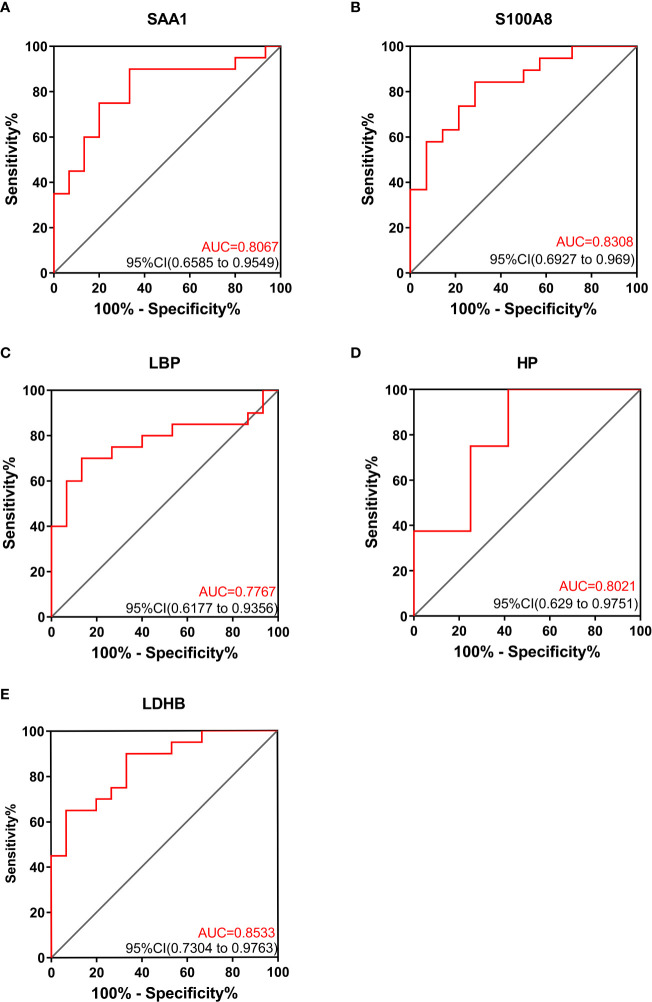
ROC curves of SAA1, S100A8, LBP, HP, LDHB between VTE group and non-VTE group.

The differentially expressed proteins were analyzed. Some of the differentially expressed proteins are fragments of immunoglobulin. Thrombosis related proteins from the upregulated proteins were selected to analyzed. SAA1 (serum amyloid A-1), S100A8 (protein S100 A8), LBP (lipopolysaccharide-binding protein), HP (haptoglobin), LDHB (lactate dehydrogenase B) were chosen. Differences in intensity of the 5 proteins between VTE group and non-VTE group are shown in **Figure 3**. The AUC of SAA1, S100A8, LBP, HP, LDHB were 0.8067, 0.8308, 0.7767, 0.8021, 0.8533, respectively (**Figure 4**).

### 3.4 Bioinformatic analysis

**Figure 5:**
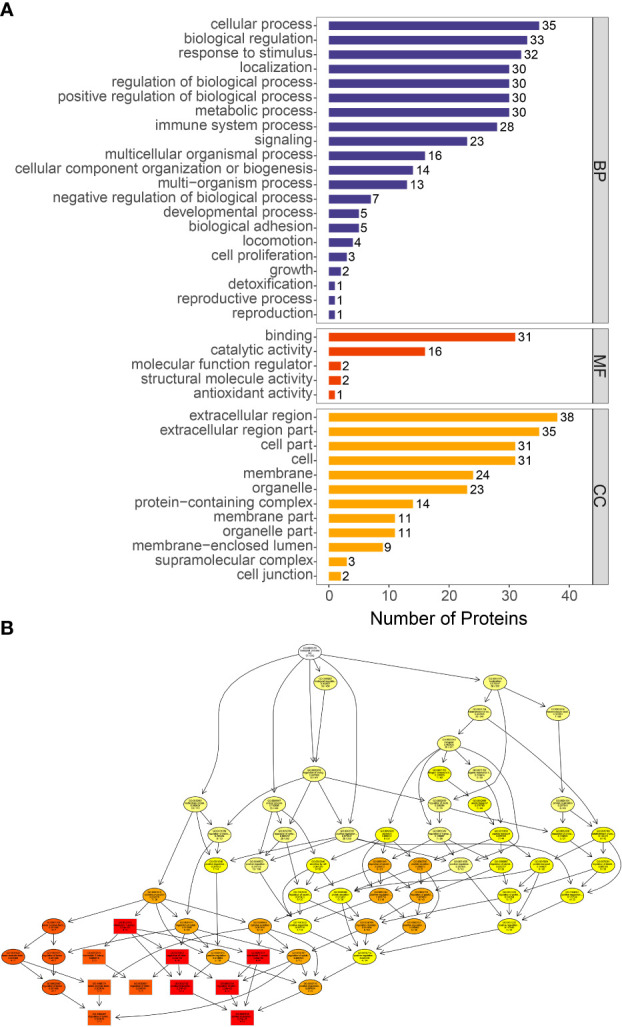
GO functional enrichment of differentially proteins. **(A)** The number of differentially expressed proteins in each categories. The abscissa represents the number of differentially expressed proteins under each functional category. **(B)** Directed acyclic graph of biological process. The branch represents the containment relationship. The lower the branch is, the more specific the function is.

We performed GO analysis of all differential proteins. GO function annotations are mainly divided into three categories: biological process, molecular function and cellular component. Through GO analysis, there are 4 biological processes with a high proportion, including acute-phase response (GO:0006953: CRP, ORM1, SAA1, LBP, ORM2, HP), cytokine production (GO:0001816: S100A8, ORM1, LBP, SAA1, immunoglobin peptides, ORM2, CRP), neutrophil migration (GO:1990266: S100A8, SAA1, LBP) (**Figure 5**).

**Figure 6:**
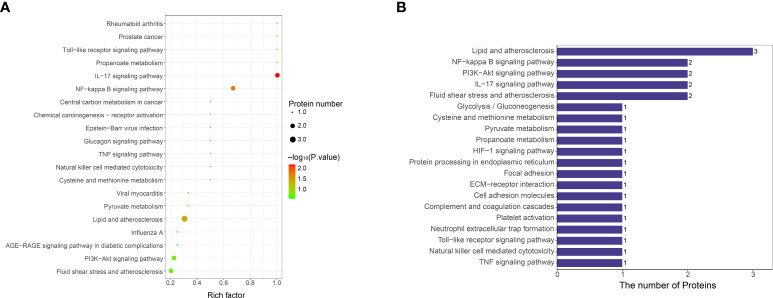
KEGG functional enrichment analysis of differentially expressed protein signaling pathways. **(A)** Bubble chart of signaling pathways associated with differentially expressed protein. The ordinate represents the statistical results of differential proteins under each KEGG pathway, the bubble color represents the significance of the enriched KEGG pathway, the P value is calculated based on Fisher’s exact test, and the color gradient represents the size of the P value (-log10), the closer the color is to red, the smaller the P value, and the higher the significance level of the corresponding metabolic pathway enrichment. **(B)** KEGG pathway annotation statistics for differentially expressed proteins. The ordinate is the name of the pathway involving differentially expressed proteins, and the abscissa represents the number of differentially expressed proteins involved in the pathway.

KEGG was used to analyze the signaling pathways of differentially expressed proteins between VTE group and non-VTE group. Differentially expressed proteins were mainly concentrated in IL-17 signaling pathway, NF-κB signaling pathway, lipid and atherosclerosis (**Figure 6**). S100A8 were mainly present in IL-17 signaling pathway. LBP were mainly present in NF-kappa B signaling pathway and lipid and atherosclerosis.

**Figure 7:**
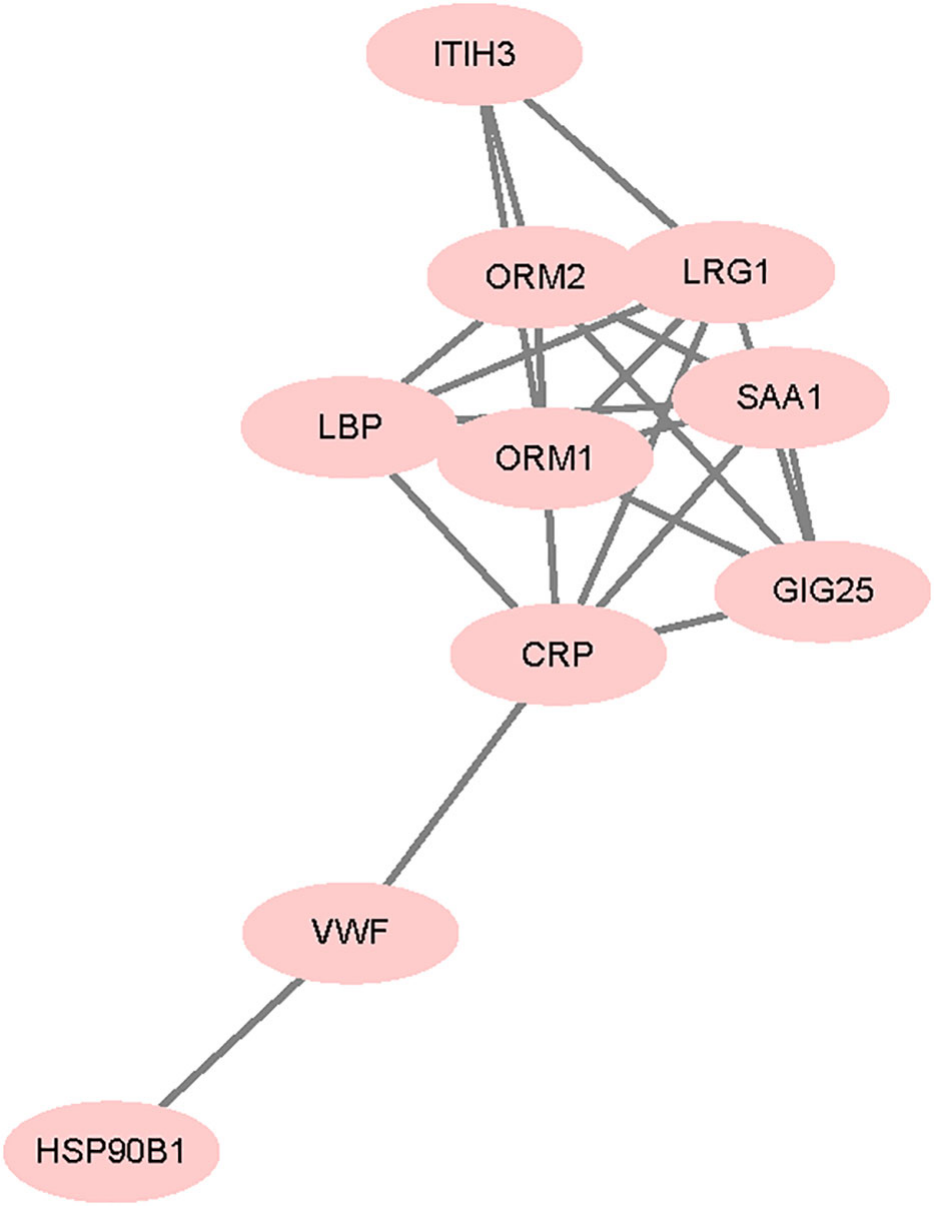
Differentially expressed protein interaction networks. Nodes represent proteins and lines represent protein-protein interactions.

Protein interaction network for the differentially expressed proteins between VTE group and non-VTE group is shown in **Figure 7**. From the 280 differentially expressed proteins, 11 proteins were involved in protein interactions. SAA1, LBP involved interacting proteins.

## 4 Discussion

In recent years, with the number of long-term cancer survivors steadily increasing, VTE in cancer patients has become a major health problem. The developments in proteomic approaches have enabled the discovery of disease-specific molecular markers, which can improve disease diagnosis and prognosis. To better understand the risk factors of VTE in NSCLC, we identified a matched control cohort of NSCLC but without VTE at the same time in the same hospital. Patients in the two groups were diagnosed with NSCLC within 3 year, which ensure that the VTE group and non-VTE group have the same background except VTE.

In this study, 42 plasma proteins were identified for upregulated in VTE group. Some of them are acute phase reactive proteins. Serum amyloid A1 (SAA1) was significantly increased in VTE group. SAA1 is a member of the SAA family which is whose level rise dramatically after trauma, infection and other stimuli. Not only is SAA a clinically useful marker of pro-inflammatory, it is considered a significant cardiovascular risk factor and clearly demonstrated to contribute to atherosclerosis development (21). SAA chemoattracts neutrophils, promotes endothelial dysfunction, induces tissue factor and ultimately activates coagulation both in arteries and veins. High levels of SAA were found to be associated with VTE and is considered to reflect tissue disturbances and inflammation associated with NSCLC invasion and metastasis, suggesting that it is a potential risk factor for VTE in NSCLC (16, 22–24).

S100A8, a member of the S100 family that normally forms a heterodimeric with S100A9 (calprotectin or S100A8/A9), was another upregulated protein in our study. Similar to SAA1, extracellular S100A8/A9 stimulates leukocyte recruitment and cytokine secretion (25). S100A8/A9 can promote tumor cell proliferation, invasion and metastasis, and its expression may be a potential prognostic factor of NSCLC patients (26). Increased S100A8/A9 were found to be associated with thrombus formation in acute coronary syndrome (ACS) (27). Kawano et al. (28) designed a S100A9 vaccine, which can result in long-term inhibition of thrombus formation through inhibition of increased S100A9/CD36 signaling without risk of bleeding or adverse autoimmune responses. Combining our findings, we thought S100A8/A9 may be a novel biomarker and therapeutic target of VTE in NSCLC patients.

Lipopolysaccharide-binding protein (LBP) binds lipopolysaccharide in the blood and transfers lipopolysaccharide to cellular receptor, which activates immune cells by producing inflammatory cytokines. Several studies have reported that LBP is a significant and independent predictor of atherosclerosis and cardiovascular disease (29–31). The pro-inflammatory effects of LBP might be an important contributor to the progression of atherosclerotic plaques. In addition to arterial disease, high LBP was found in fibrin clot of acute PE patients (32). Considering the data shown in this study, we speculate that LBP may promote vascular inflammation in NSCLC patients with VTE.

Reactive oxygen has been shown to cause endothelial damage, to promote atherosclerosis and to favor the development of thrombotic diseases. Haptoglobin (HP) is a plasma protein that binds free hemoglobin and inhibits the production of reactive oxygen species. High levels of serum HP were found to predict atherothrombotic (33). The study of Vormittag et al. (34) demonstrated that HP2-2, a kind of genotype of HP, is a risk factor for spontaneous VTE, presumably through a pathophysiological mechanism similar to arterial disease. Lactate dehydrogenase (LDH) is a tetramer composed of LDHA and LDHB subunits, which is most often measured to evaluate tissue damage. LDHA is particularly abundant in skeletal muscle and liver while LDHB is the major form in cardiac muscle. Elevated LDH levels is currently accepted as an important metric when there is clinical suspicion of pump induced thrombosis, as this kind of disorders are often accompanied by hemolysis (35, 36). LDHB and HP were elevated in the VTE group, indicating that NSCLC patients with VTE may have hemolysis or tissue damage.

The use of LMWH in VTE group was before blood collection in this study. However, LMWH have been applied for anti-inflammatory besides anticoagulation. Our results still showed a higher inflammatory state in VTE group than non-VTE group (37, 38). Previous studies have also suggested that patients with acute or chronic inflammation are at high risk of VTE. Inflammatory mediators can cause the expression of tissue factor, increase the levels of fibrinogen and plasminogen activator inhibitor, thus initiating the coagulation cascade and suppressing the fibrinolytic system (39).

Interestingly, the differentially expressed proteins we identified are not only associated with VTE, but also risk factors for arterial thrombosis. Arterial thrombosis and VTE were regarded as two completely different diseases in the past. Recent studies indicated that the two diseases share common risk factors, pathological mechanisms and preventive measures (40–42). Inflammation is most likely the underlying pathogenesis and the common mechanism by which different risk factors trigger venous and arterial thrombosis (43). Sussman et al. (44) found that most of the highly overexpressed genes in lung cancer with VTE were consistent with those identified in arterial thrombosis and inflammatory responses, which are quite consistent with the conclusion drawn by our proteomics analysis.

The blood routine parameters results confirmed DIA-MS analysis. Our clinical data show that neutrophil to lymphocyte ratio (NLR) in VTE group is significantly higher than non-VTE group (5.95 vs 2.40, *P*=0.002), which indicate that there is activation of inflammatory response in VTE group. Patients with lung cancer often exhibit leukocytosis (45). Activated neutrophils could enhance thrombosis in cancer patients by releasing neutrophil extracellular traps (NETs) (6). Murine models have confirmed that NETs contribute to thrombosis in lung cancer (46). Lung cancer patients with high NLR (≥3) also had a lower chance of VTE resolution (47). RBC and HGB in VTE group were significantly lower than those in non-VTE group. Decreased HGB are useful for predicting VTE risk in NSCLC patients (48), and prechemotherapy HGB level < 100 g/L is one of the laboratory parameters of VTE risk assessment in cancer patients (Khorana score) (49). The mechanism is less clear, possibly due to damage. In contrast, PDW was high in VTE group. PDW indicates variation in platelet size. Activated platelets provide procoagulant surface and release multiple molecules to amplify coagulation process. As an early index of platelet activation, increased PDW has been observed to be associated with artery thrombosis and VTE in many diseases (50–52). The role of PDW in NSCLC related VTE remains to be further confirmed.

The results of the bioinformatic analysis proved that inflammation, repair and cytokine play important roles in promoting the development of VTE and VTE may share common risk factors and pathways with arterial thrombosis in NSCLC, which may be useful for further investigations.

Nevertheless, there were some limitations in this study. First, the number of samples was small. Also, the identified differential proteins should be validated by other methods in future study and deep investigations are needed on the relevant mechanisms. To our knowledge, this study is the first to employ proteomics profiling to identify differentially expressed proteins and possible pathways for VTE in NSCLC patients, which may provide insights to develop new biomarkers for diagnosis, risk stratification and treatment of VTE in lung cancer.

## Data availability statement

The datasets presented in this study can be found in online repositories. The names of the repository/repositories and accession number(s) can be found below: http://www.proteomexchange.org/, PXD037222.

## Ethics statement

Written informed consent was obtained from the individual(s) for the publication of any potentially identifiable images or data included in this article.

## Author contributions

LG conceived and designed the experiments. YL conducted the experiments and data analysis. Material preparation was performed by RM and GC. YA and YX provided technical support of experiments. YL and LG wrote the manuscript. YF prepared the figures and critically revised the manuscript. All authors contributed to the article and approved the submitted version.
